# YOLOv11-MPD: A Multi-Part Maize Disease Detection Algorithm for Complex Field Environments

**DOI:** 10.3390/s26175383

**Published:** 2026-08-26

**Authors:** Rui Dong, Longtao Jin, Zhaozhao Cai

**Affiliations:** 1 College of Computer and Information Engineering, Xinjiang Agricultural University, Urumqi 830052, China; 17536010624@163.com (R.D.); ltj17436@gmail.com (L.J.); 2 Xinjiang Engineering Technology Research Center for Agricultural Informatization, Urumqi 830052, China

**Keywords:** maize disease detection, YOLOv11n, YOLOv11-MPD, object detection, feature enhancement, multi-scale feature fusion

## Abstract

Maize diseases affecting leaves, stalks, and ears can substantially reduce yield and quality; therefore, rapid and accurate recognition in complex field environments is important for intelligent agricultural monitoring. To address the large-scale variation, weak fine-grained texture, and strong background interference associated with multi-part maize diseases, this study proposes YOLOv11-MPD (YOLOv11 for Maize Multi-Part Disease Detection), a maize disease detection algorithm based on YOLOv11n. The method jointly improves spatial position awareness, shallow detail preservation, local-context modeling, key semantic-region enhancement, and lightweight detection-head reconstruction. RFCAConv, C3k2_RFCAConv, and Detect_LSDECD are introduced into the baseline network to strengthen directional texture modeling, multi-scale feature aggregation, and detection-head feature representation. FG-RFCAConv, HGD-C3k2, LCA-C3k2, and GRN-BiAttn are further designed for high-frequency differential gated detail compensation, P3 high-resolution detail enhancement, local-context fusion, and global-response-normalized attention regulation, respectively. Experimental results show that YOLOv11-MPD achieves Precision, Recall, mAP50, and mAP50-95 of 72.3%, 72.8%, 79.5%, and 50.2%, improving YOLOv11n by 2.4, 2.5, 2.9, and 2.4 percentage points, respectively, while reducing parameters from 2.6 M to 2.4 M. These results indicate that, within the scope of the dataset used in this study, YOLOv11-MPD improves multi-part maize disease detection under complex field conditions. However, the current conclusions are limited to the constructed dataset, and further validation using larger multi-region, multi-season, and multi-device datasets is required to evaluate its broader generalization ability.

## 1. Introduction

Crop diseases are among the major factors affecting crop yield, quality, and agricultural economic benefits. Rapid and accurate identification of diseased areas is an essential basis for precision prevention and control as well as smart agriculture [1]. As an important food, feed, and industrial raw-material crop, maize is closely related to food security and livestock production [2]. During growth, however, maize is susceptible to leaf spot, gray leaf spot, rust, mosaic, stalk rot, and ear diseases. These diseases can reduce leaf photosynthesis, weaken stalk resistance, and hinder ear development, thereby affecting maize yield and quality [3,4,5,6]. Therefore, intelligent recognition of multi-part maize diseases in field environments is significant for early warning, precision spraying, and green disease control.

Traditional disease recognition mainly relies on manual field scouting and empirical judgment, which are subjective, inefficient, poorly real time, and difficult to scale to large-area continuous monitoring [7]. With the development of computer vision and deep learning, convolutional neural networks can automatically extract color, texture, morphology, and spatial semantic features from images, and have been widely applied to crop disease classification, object detection, and diseased-region localization [8,9]. Among object detection methods, two-stage algorithms such as Faster R-CNN have strong candidate-region representation capability but relatively complex structures; one-stage algorithms such as SSD and the YOLO series have advantages in detection speed, end-to-end prediction, and deployment convenience, and have gradually become common methods for agricultural object detection [10,11,12].

In recent years, extensive studies on crop disease detection under complex field environments have focused on multi-scale feature fusion, attention mechanisms, lightweight networks, and detection-head optimization. Sun et al. [13] proposed a multi-scale self-attention feature fusion method for tea diseases with variable scales, dense distributions, and occlusion, improving disease feature representation. Ma et al. [14] introduced a lightweight backbone, an attention mechanism, and a feature reorganization module into YOLOv8 to achieve lightweight wheat disease detection. Bai et al. [15] improved the downsampling structure, attention mechanism, and multi-scale fusion network of YOLOv8n to enhance peanut leaf disease detection. In addition, studies on diseases or pest-disease detection in apple, tea, ginger, wheat, and tomato have shown that improved YOLO-series models can strengthen recognition of small lesions and diseases with similar textures under complex backgrounds [16,17,18,19,20,21,22,23]. However, these studies mainly improve detection performance from a specific perspective, such as feature fusion, attention enhancement, lightweight backbone design, or detection-head optimization. Although such single-aspect improvements are effective for certain crop disease scenarios, they may be insufficient for multi-part maize disease detection, where disease targets differ markedly in organ location, lesion scale, texture morphology, boundary clarity, and background interference. Therefore, a more coordinated architectural design is required to jointly enhance feature extraction, multi-scale feature fusion, and detection prediction.

For maize-related target recognition, existing studies mainly focus on maize leaf diseases or maize root stubble. Yang et al. [24] detected maize root stubble using an improved YOLOv11n, indicating that the YOLOv11 series has application potential for agricultural target recognition in complex backgrounds. Sun et al. [25] studied Blight detection under complex field environments, providing a reference for maize disease object detection. Nevertheless, most existing maize disease detection studies focus on leaf disease recognition, while unified detection of diseases on maize leaves, stalks, and ears has received insufficient attention. Compared with a single leaf disease, multi-part maize diseases are more complex in target scale, texture morphology, spatial distribution, and background interference, which can lead to missed detection of small lesions, inaccurate boundary localization, and false detection caused by similar textures. These challenges are coupled across different stages of the detection network. Small and weak lesions require stronger shallow detail preservation during feature extraction, large-scale variation requires effective multi-scale feature fusion, complex background interference requires more discriminative semantic-region enhancement, and accurate localization requires a more consistent detection-head representation. Therefore, modifying only one architectural component is unlikely to fully address these problems simultaneously.

To address these issues, this study takes YOLOv11n as the baseline and proposes YOLOv11-MPD for detecting maize diseases on leaves, stalks, and ears. Unlike methods that optimize only a single network component, YOLOv11-MPD performs coordinated optimization across feature extraction, feature fusion, semantic-region enhancement, and detection prediction. The model is jointly designed around spatial position awareness, shallow detail preservation, local-context modeling, multi-scale feature fusion, key semantic-region enhancement, and lightweight detection-head reconstruction, aiming to improve the network adaptability to small weak lesions, blurred boundaries, fine-grained textures, and complex background interference.

The main contributions of this study are as follows:

(1) A hybrid multi-part maize disease dataset composed of self-collected field images and public data was constructed, providing a data basis for detecting maize leaf, stalk, and ear diseases in complex scenes.
(2) A YOLOv11n-based multi-part maize disease detection model, YOLOv11-MPD, was proposed. Through spatial position awareness, shallow detail preservation, local-context modeling, and shared detection-head enhancement, the model improves the representation of multi-scale lesions, weak texture boundaries, and diseased regions under complex backgrounds.
(3) Comparative experiments, ablation experiments, qualitative visualization, training-curve visualization, and Raspberry Pi 5-based edge deployment experiments were conducted to evaluate the performance of the proposed method on the multi-part maize disease dataset constructed in this study.

## 2. Materials and Methods

### 2.1. Dataset Collection and Preprocessing

**Figure 1:**
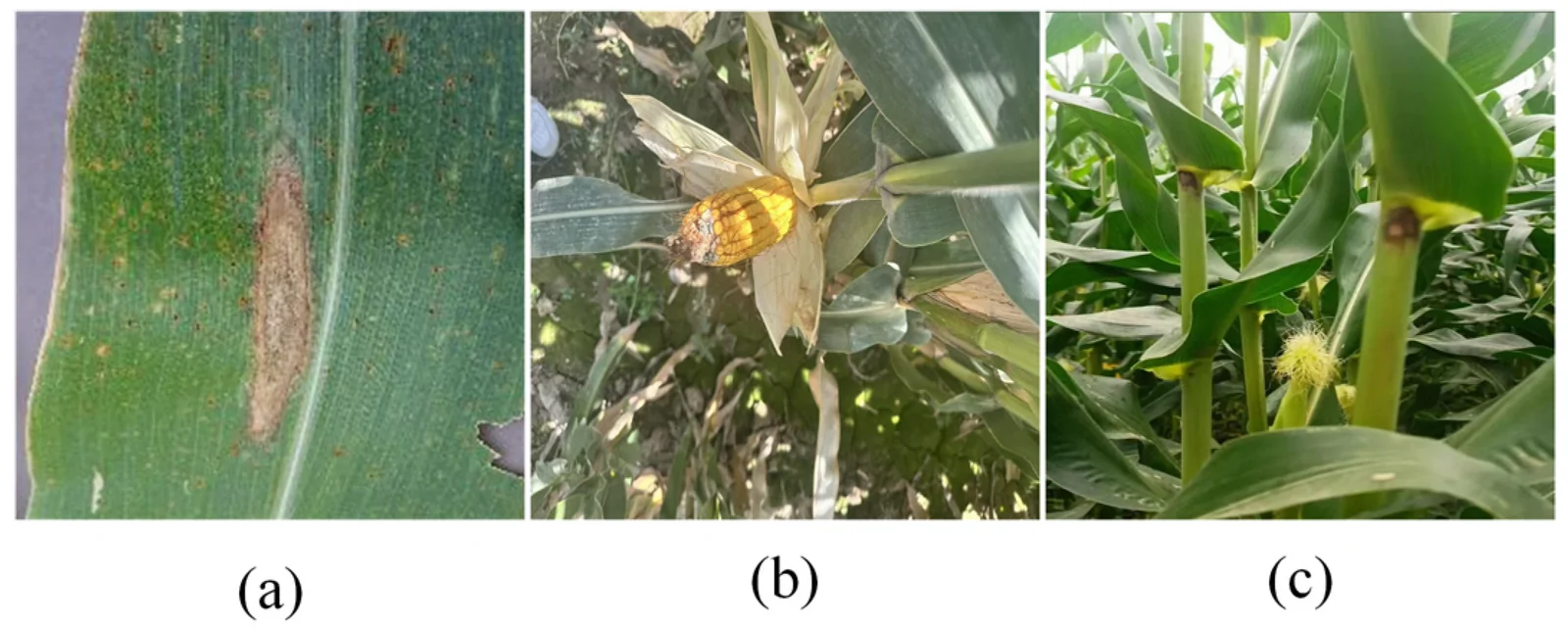
Example images from the maize disease dataset. (**a**) Northern corn leaf blight; (**b**) Ear_rot; (**c**) stalk_rot.

This study constructed a multi-source image dataset containing three maize diseases: Blight, Ear_rot, and Stalk_rot. First, images of Blight were obtained from the public Kaggle consolidated-corn-dataset, and samples with clear lesions and good image quality were retained after manual screening. Second, Ear_rot and Stalk_rot images were collected at an agricultural experimental station in Xinjiang, China. Field images were captured using a vivo X21 smartphone. All selected images exhibited clear and typical late-stage disease symptoms; therefore, lesion severity was relatively consistent across the dataset, and no further severity stratification was performed. In this study, complex field environments mainly refer to variations in illumination, shooting distance, and viewing angle, as well as interference from natural backgrounds such as leaves, stalks, and soil. Examples of maize disease characteristics are shown in Figure 1.

**Figure 2:**
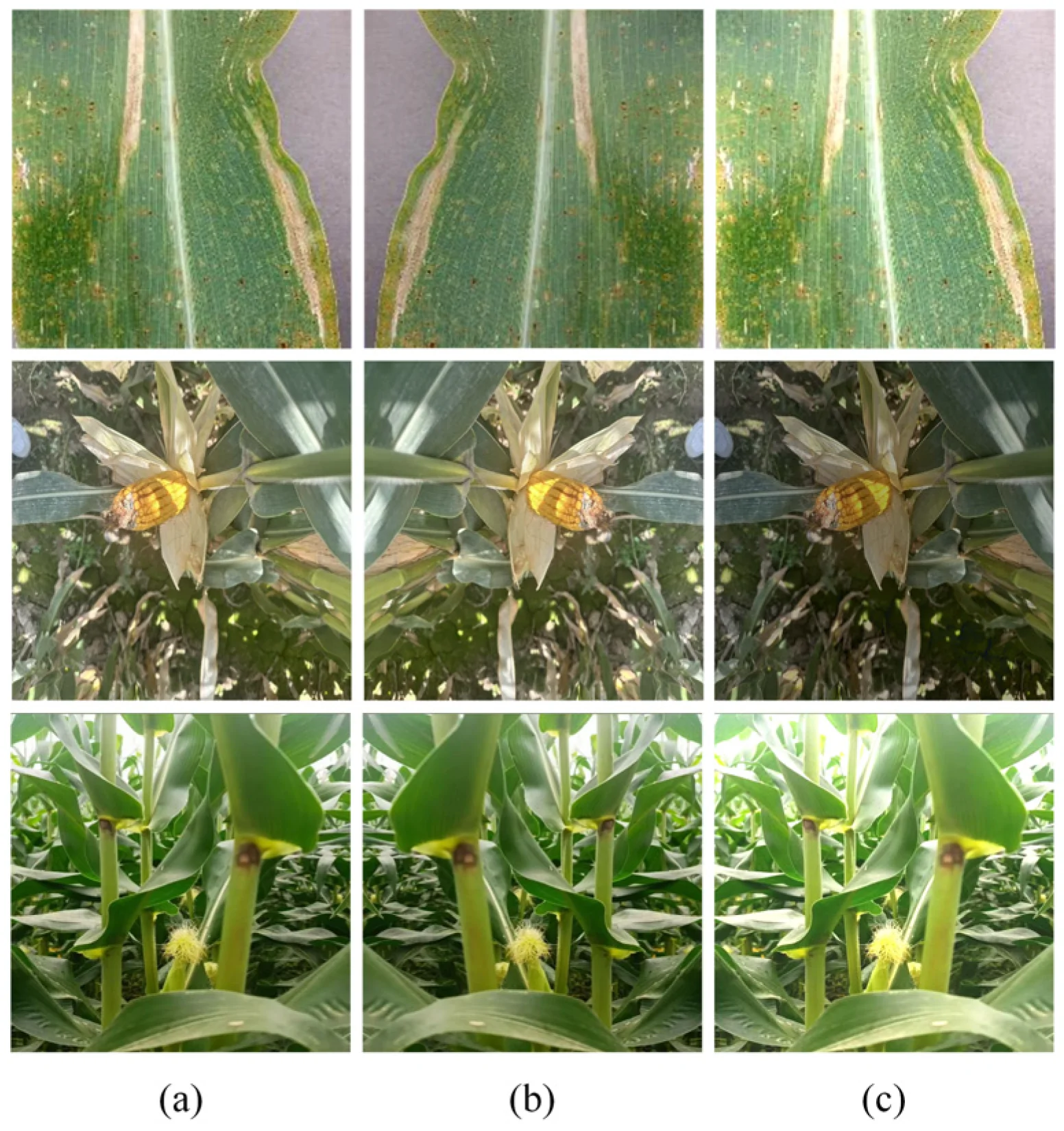
Examples of data augmentation. (**a**) Original images; (**b**) horizontally flipped images; (**c**) brightness-adjusted images.

The selected original images were then divided into training, validation, and test sets at a ratio of 8:1:1. Data splitting was performed before data augmentation to avoid information leakage between the training, validation, and test sets. To further expand the training samples and simulate the effects of shooting direction and illumination changes on image quality in natural environments, data augmentation was applied only to the training images; the validation and test sets were not augmented. The augmentation methods included mirror flipping and brightness variation, as shown in Figure 2. Finally, maize disease regions were manually annotated using X-AnyLabeling-CPU, and the annotations were saved in YOLO format. The detailed dataset statistics after data splitting and training-set augmentation are summarized in Table 1.

### 2.2. Baseline YOLOv11n Model

**Figure 3:**
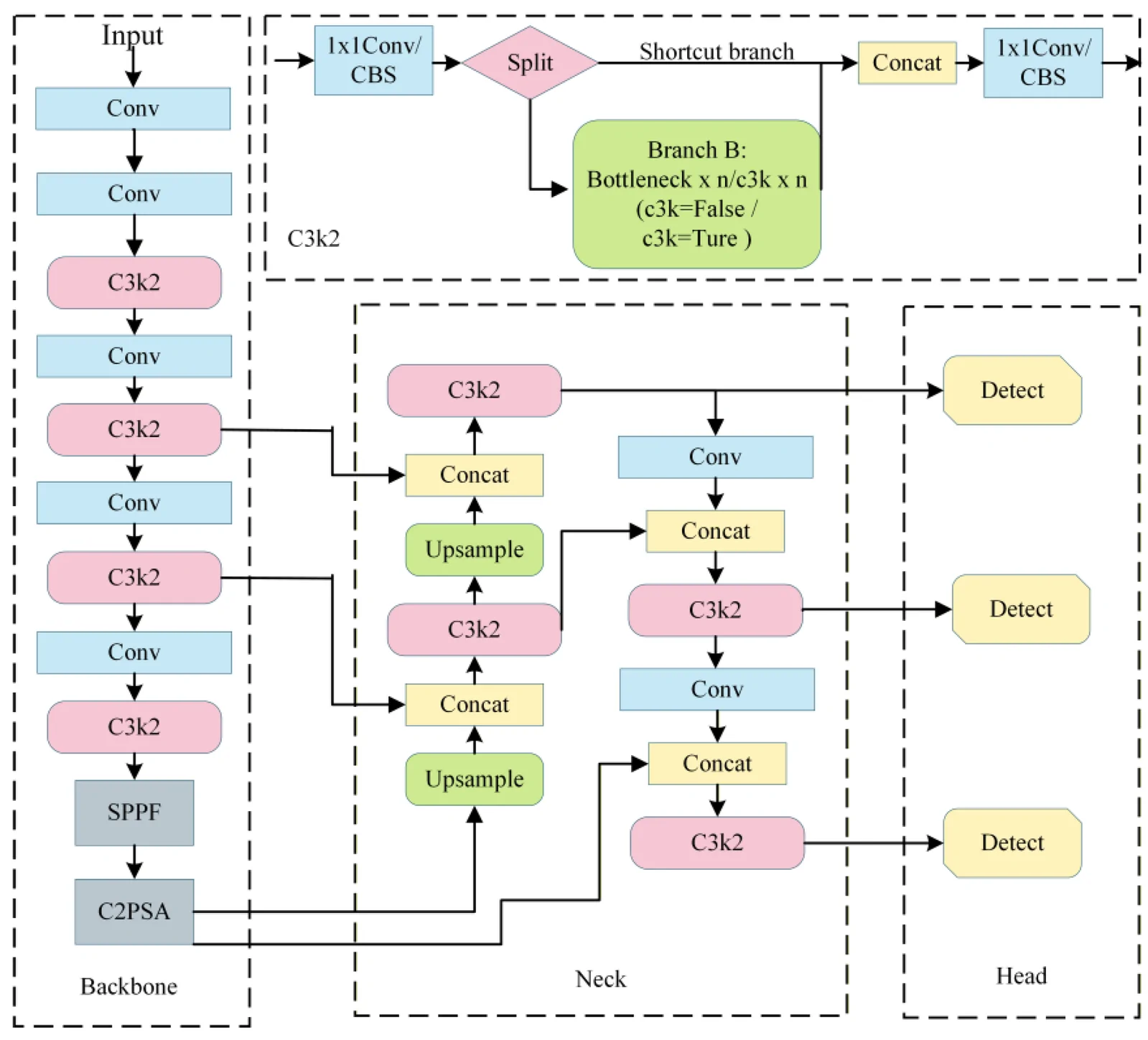
Structure of the YOLOv11n model.

YOLOv11n belongs to the YOLOv11 model family and consists of a backbone, a neck, and a detection head [26]. Its structure is shown in Figure 3. The backbone extracts hierarchical features using Conv, C3k2, SPPF, and C2PSA modules; the neck fuses P3, P4, and P5 multi-scale features to enhance target feature representation; and the detection head predicts object categories and regresses bounding boxes. YOLOv11n provides strong feature extraction capability and stable training, making it suitable for detection tasks on medium- and small-scale maize disease datasets.

In multi-part maize disease recognition, disease targets are distributed across leaves, stalks, and ears, with large-scale differences, weak texture details, obvious morphological variation, and strong background interference. Although YOLOv11n has good general object detection capability, it still faces insufficient fine-grained lesion feature extraction, inadequate multi-scale target representation, and false or missed detections in complex field environments. Therefore, it is necessary to optimize the feature extraction structure, multi-scale feature fusion strategy, and detection-head design based on YOLOv11n to improve detection accuracy and robustness for multi-part maize disease targets.

### 2.3. YOLOv11-MPD

**Figure 4:**
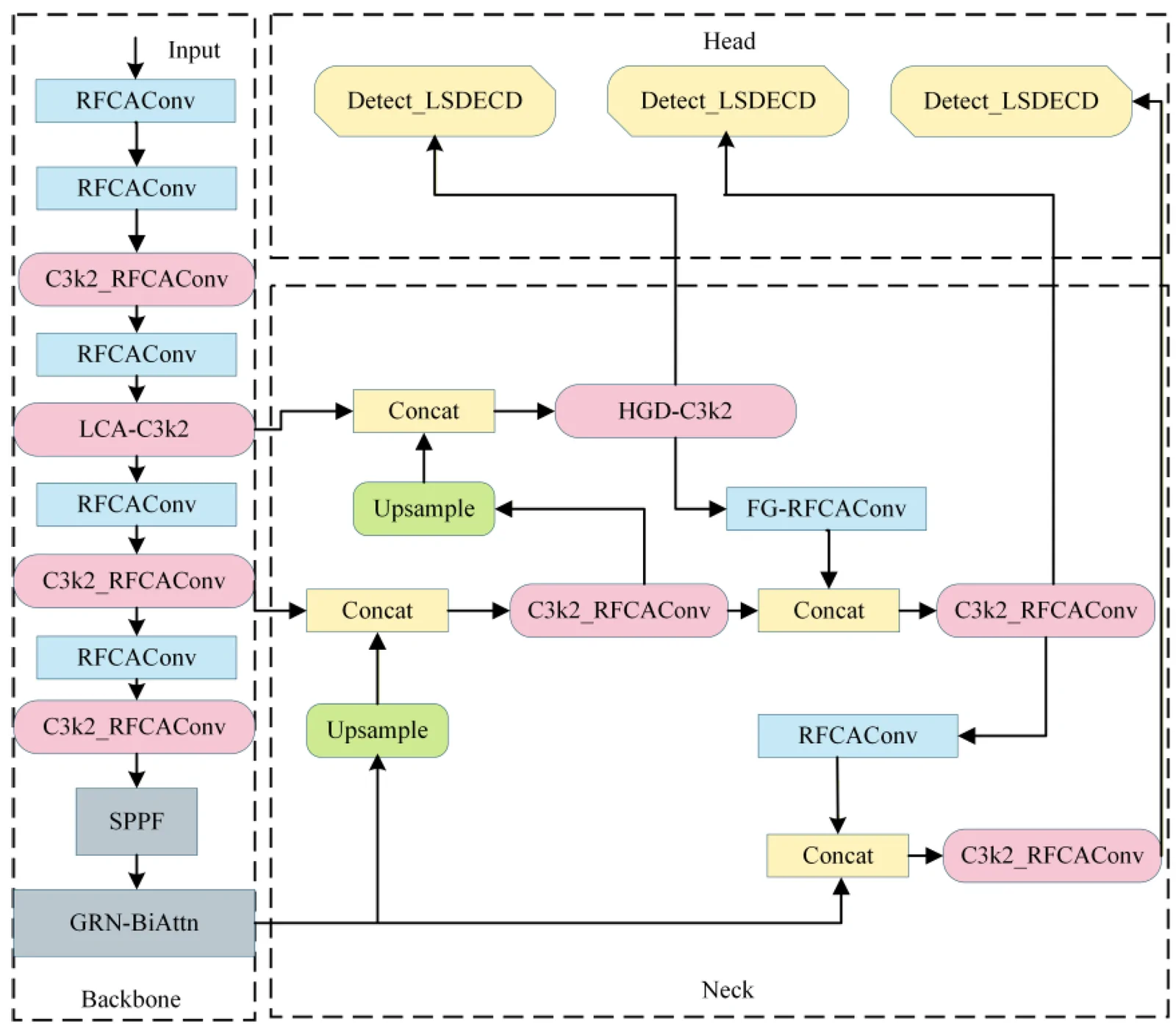
Structure of the YOLOv11-MPD model.

To address the limitations of YOLOv11n in multi-part maize disease detection, this study constructs YOLOv11-MPD, whose overall structure is shown in Figure 4. The model uses YOLOv11n as the baseline framework and introduces targeted improvements to feature extraction, feature fusion, and detection output according to the morphological, scale, and texture distribution differences among leaf, stalk, and ear diseases, thereby improving perception of diseased regions on different maize parts.

The architectural design of YOLOv11-MPD follows a unified task-driven concept rather than a simple combination of independent modules. For maize diseases occurring on leaves, stalks, and ears under complex field environments, the main detection difficulties are coupled across multiple network stages, including large target-scale variation, weak lesion texture, blurred disease boundaries, and strong background interference. Therefore, the proposed model is designed to improve disease feature representation from three coordinated levels: feature extraction, feature fusion, and detection prediction. In this framework, shallow and middle feature extraction modules enhance lesion texture and spatial-position responses, feature fusion modules preserve high-resolution details and compensate for information loss during scale transfer, and the detection head improves the consistency of multi-scale prediction features. This coordinated design aims to form a unified architecture for multi-scale, weak-texture, and complex-background maize disease detection.

Specifically, in the feature extraction stage, RFCAConv and C3k2_RFCAConv enhance lesion spatial position and directional texture representation, while LCA-C3k2 and GRN-BiAttn further strengthen local-context information interaction and high-level semantic-region selection. In the feature fusion stage, HGD-C3k2 and FG-RFCAConv are designed to enhance fine-grained lesion features in the P3 branch and compensate for edge and texture information loss during P3-to-P4 scale transfer, respectively. In the detection output stage, Detect_LSDECD performs shared detail enhancement on multi-scale detection features to improve disease target classification and localization. Through these coordinated structures, YOLOv11-MPD is designed to address large-scale differences, weak boundary textures, and complex background interference in multi-part maize disease detection.

### 2.4. Introduced Baseline Modules

#### 2.4.1. RFCAConv Module

**Figure 5:**
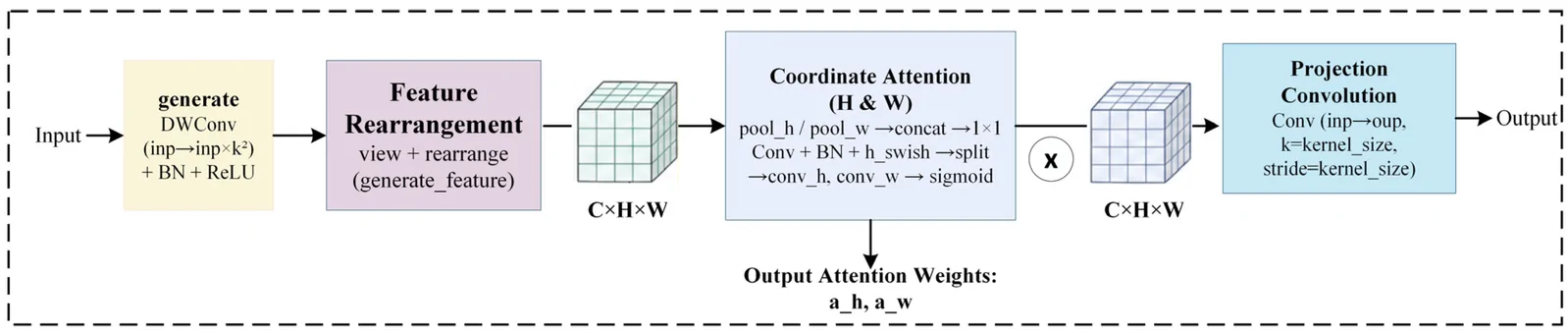
Structure of the RFCAConv module. The symbol “⊗” denotes element-wise multiplication.

In complex field environments, maize lesion areas are easily disturbed by leaf veins, stalk textures, and background vegetation. Standard convolution mainly relies on local sliding windows for feature extraction and has limited ability to selectively model directional textures and spatial position relationships. RFCAConv strengthens texture responses and positional constraints in different directions through receptive-field feature rearrangement and coordinate attention modeling along height and width, and it has been applied in agricultural vision tasks [27]. Accordingly, this study introduces RFCAConv to replace selected Conv modules in YOLOv11n, as shown in Figure 5. In the proposed model, RFCAConv mainly acts in shallow and middle feature extraction stages to enhance lesion edges, texture orientation, and spatial position responses, thereby improving perception of multi-part maize disease regions under complex backgrounds.

#### 2.4.2. C3k2_RFCAConv Module

**Figure 6:**
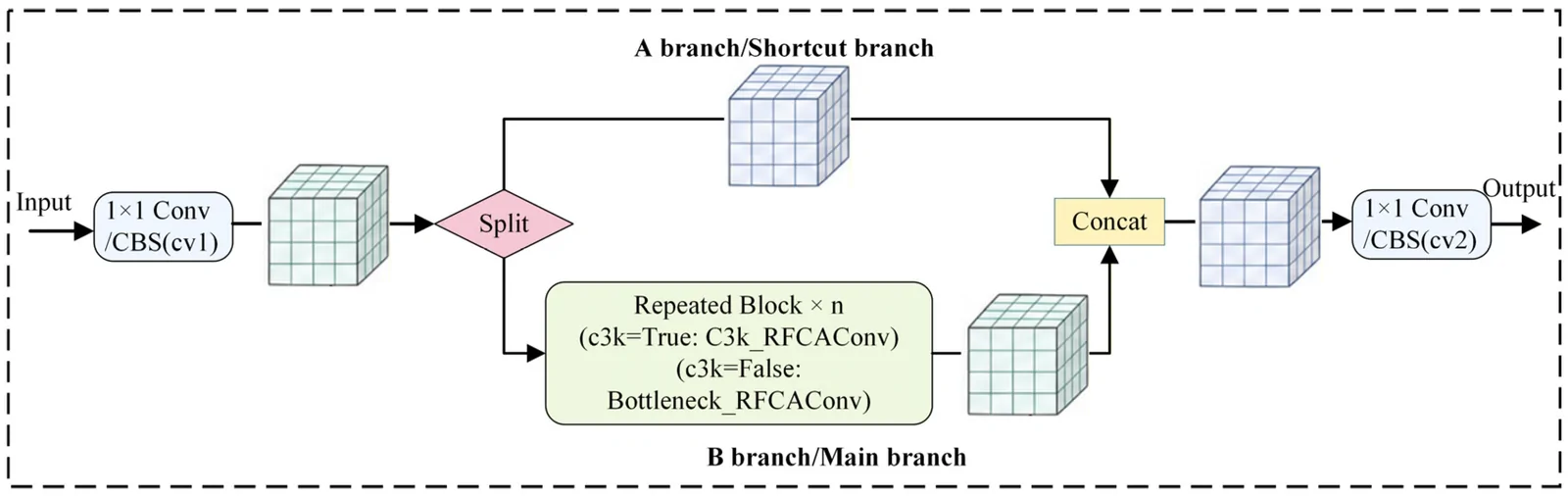
Structure of the C3k2_RFCAConv module.

Maize leaf, stalk, and ear diseases differ substantially in morphology, scale, edge clarity, and texture distribution. Although the C3k2 module in YOLOv11n can aggregate cross-stage features, its modeling of directional position relationships for weak-texture lesions and irregular boundaries remains limited. To improve fine-grained structural representation of diseased regions, this study introduces C3k2_RFCAConv to replace selected C3k2 modules in YOLOv11 [28], as shown in Figure 6. C3k2_RFCAConv embeds RFCAConv feature extraction units into the C3k2 feature fusion framework and converts the original internal feature extraction branch into an enhanced branch with receptive-field rearrangement and coordinate directional attention, allowing the model to incorporate directional texture and spatial position constraints while aggregating features.

#### 2.4.3. Detect_LSDECD Detection Head

**Figure 7:**
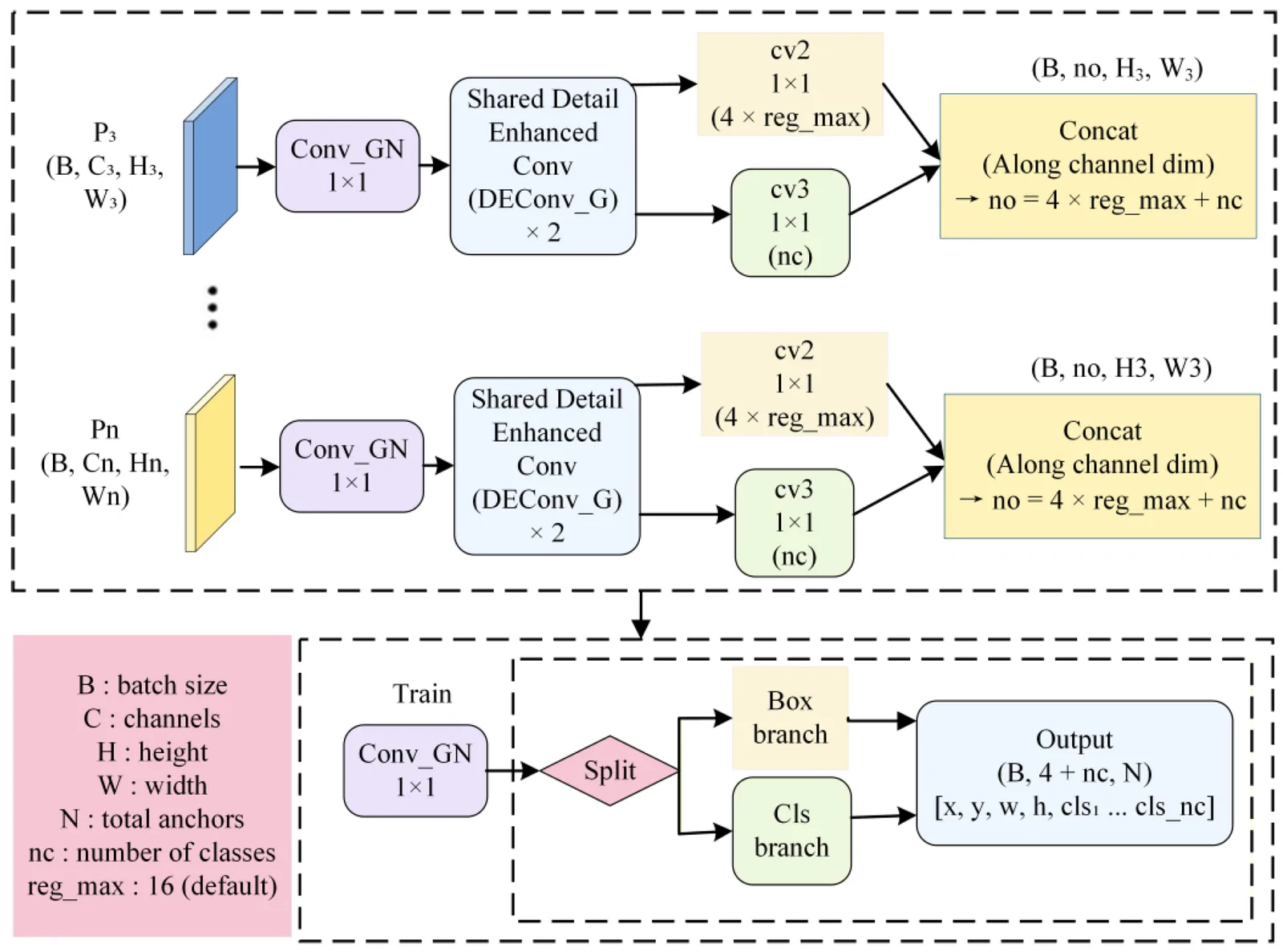
Structure of the Detect_LSDECD module.

The original Detect head of YOLOv11n performs feature transformation and prediction separately on P3, P4, and P5, which causes repeated computation and inconsistent detail representation across scales. To enhance the use of maize disease edge and local texture information at the detection end [29], this study replaces the original Detect head with Detect_LSDECD. Its structure is shown in Figure 7. The detection head first maps features at different scales to a unified channel dimension through 1 × 1 convolution and then uses a shared detail-enhancement convolution branch to enhance multi-scale features.

In the proposed model, Detect_LSDECD retains the three-scale detection strategy of YOLOv11n. This structure improves detail representation consistency across detection scales, thereby strengthening localization of small lesions, blurred boundaries, and diseased regions under complex field backgrounds.

### 2.5. Multi-Level Collaborative Enhancement Modules

#### 2.5.1. FG-RFCAConv and HGD-C3k2 (Fine-Grained Detail-Compensation RFCAConv and High-Frequency Gated Detail-Enhancement C3k2)

**Figure 8:**
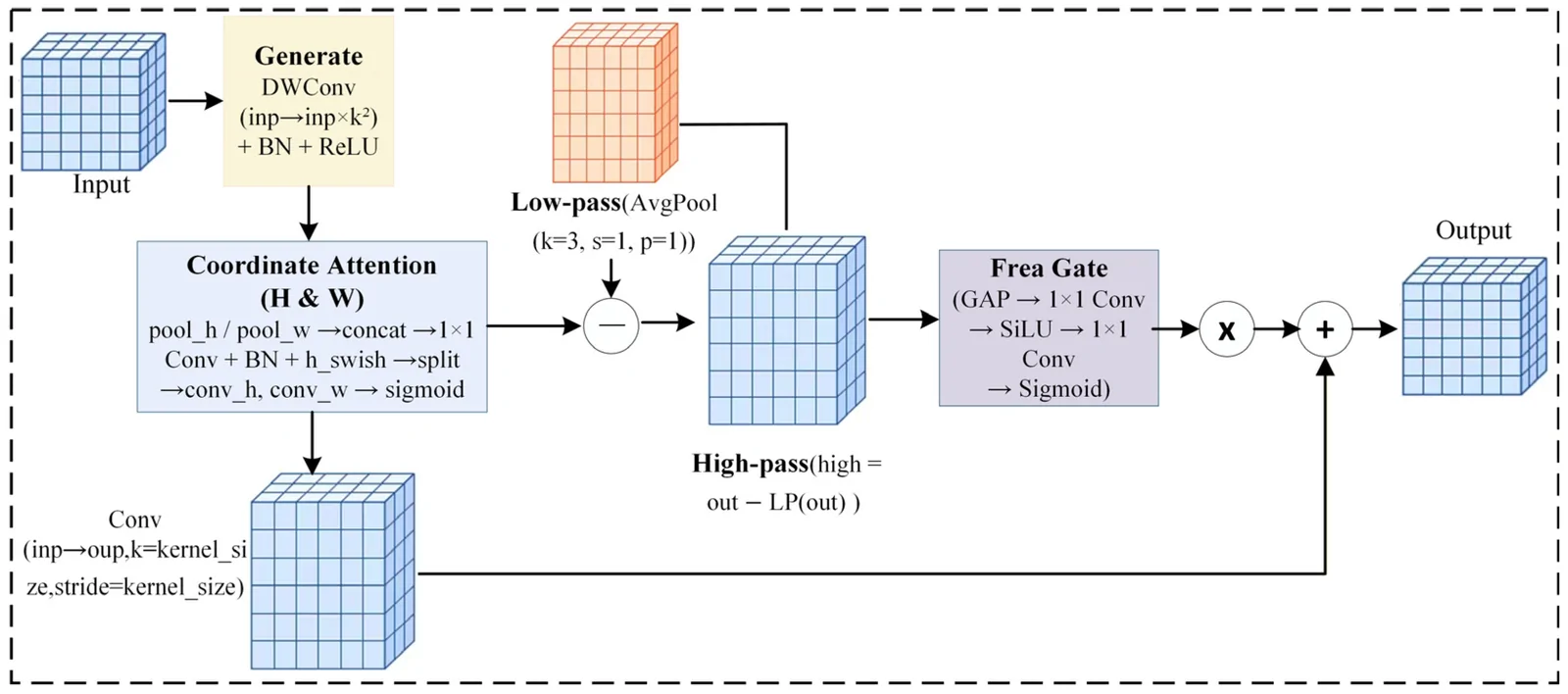
Structure of the FG-RFCAConv module. The symbols “⊖” and “⊕” denote element-wise subtraction and element-wise addition, respectively.

**Figure 9:**
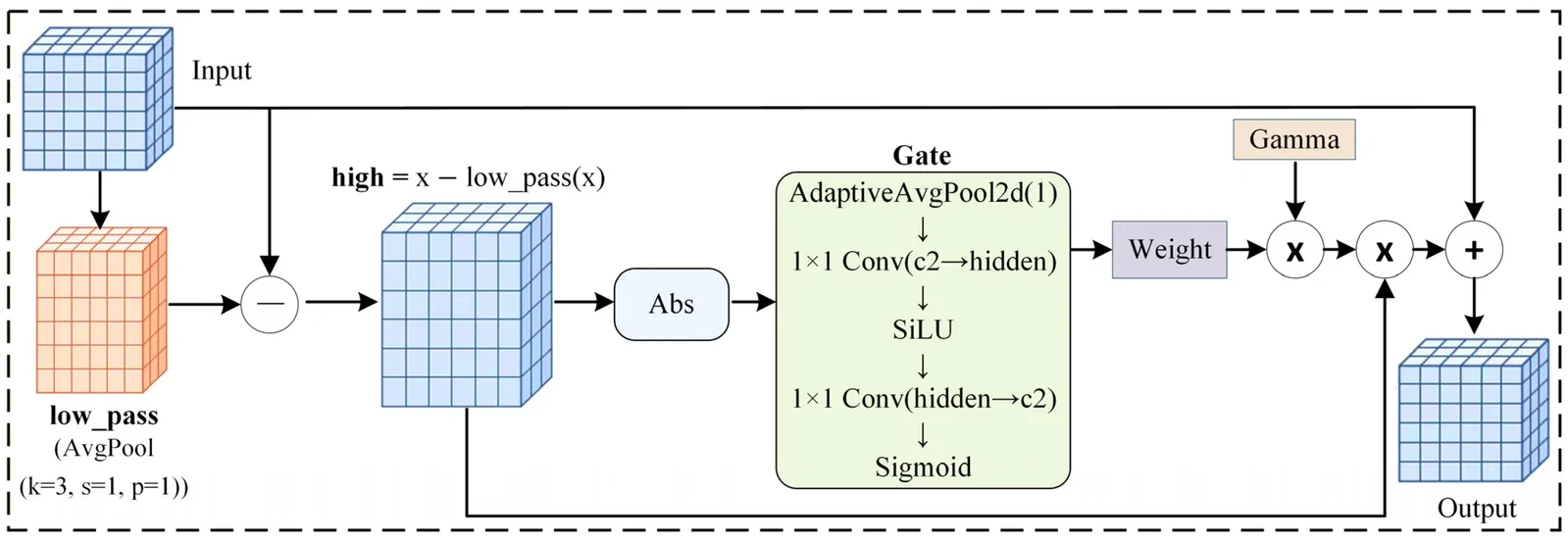
Structure of the HGD-C3k2 module.

For maize disease images with many small lesions, weak edge textures, and shallow details that are easily attenuated during scale transfer, this study designs two collaborative enhancement modules, FG-RFCAConv and HGD-C3k2, from the perspectives of high-resolution feature enhancement and scale-transfer detail compensation. Their structures are shown in Figure 8 and Figure 9, respectively. Existing small-object detection studies indicate that strengthening high-resolution feature representation and preserving detail information during scale transfer help improve small-object localization and detection stability under complex backgrounds [30,31]. Based on the feature requirements of multi-part maize disease detection, HGD-C3k2 mainly enhances shallow fine-grained texture features in the P3 branch, whereas FG-RFCAConv compensates for edge and spatial position information loss during P3-to-P4 scale conversion.

In YOLOv11n, the fused P3 feature usually passes through a C3k2 module for feature aggregation and then through a Conv module for downsampling from P3 to P4. This process weakens detail responses in small lesions, weak texture edges, and local mutation regions. Therefore, this study embeds a high-frequency differential gated detail enhancement mechanism into the P3 high-resolution feature aggregation process to construct HGD-C3k2, strengthening shallow texture representation of small lesions. The proposed high-frequency differential gated mechanism is further optimized and reconstructed to fit the P3-to-P4 downsampling feature transfer process, forming FG-RFCAConv to reduce loss of disease edge and local structural information during scale conversion. The two modules act on P3 detail enhancement and P3-to-P4 scale-transfer compensation, respectively, forming a collaborative detail-preservation strategy for small disease targets.

The high-frequency response can be expressed as:

(1) $$H=O-P_{avg}(O)$$

where H denotes the high-frequency detail feature, O denotes the output feature after directional attention weighting, and $P_{avg}$ denotes the average-pooling low-pass filtering operation.

To suppress background noise interference during high-frequency enhancement, the module further uses a high-frequency differential gating branch to adaptively weight the high-frequency response and controls the enhancement intensity through learnable parameters. The output feature is expressed as:

(2) Y=O+tanh(γ)⋅H⋅G(|H|)

where Y denotes the final output feature of the module, G denotes the high-frequency differential gating function composed of global average pooling, convolutional mapping, and a sigmoid function, |H| denotes the absolute response of the high-frequency feature, and γ is a learnable scaling parameter.

HGD-C3k2 strengthens shallow texture representation in the P3 small-target branch, while FG-RFCAConv improves edge detail preservation during feature transfer from P3 to P4. Together, they form a collaborative detail enhancement strategy for P3 small-scale disease targets, aiming to improve the representation of maize leaf, stalk, and ear disease features.

#### 2.5.2. LCA-C

##### 3k2 (Local-Context Adaptive C3k2)

**Figure 10:**
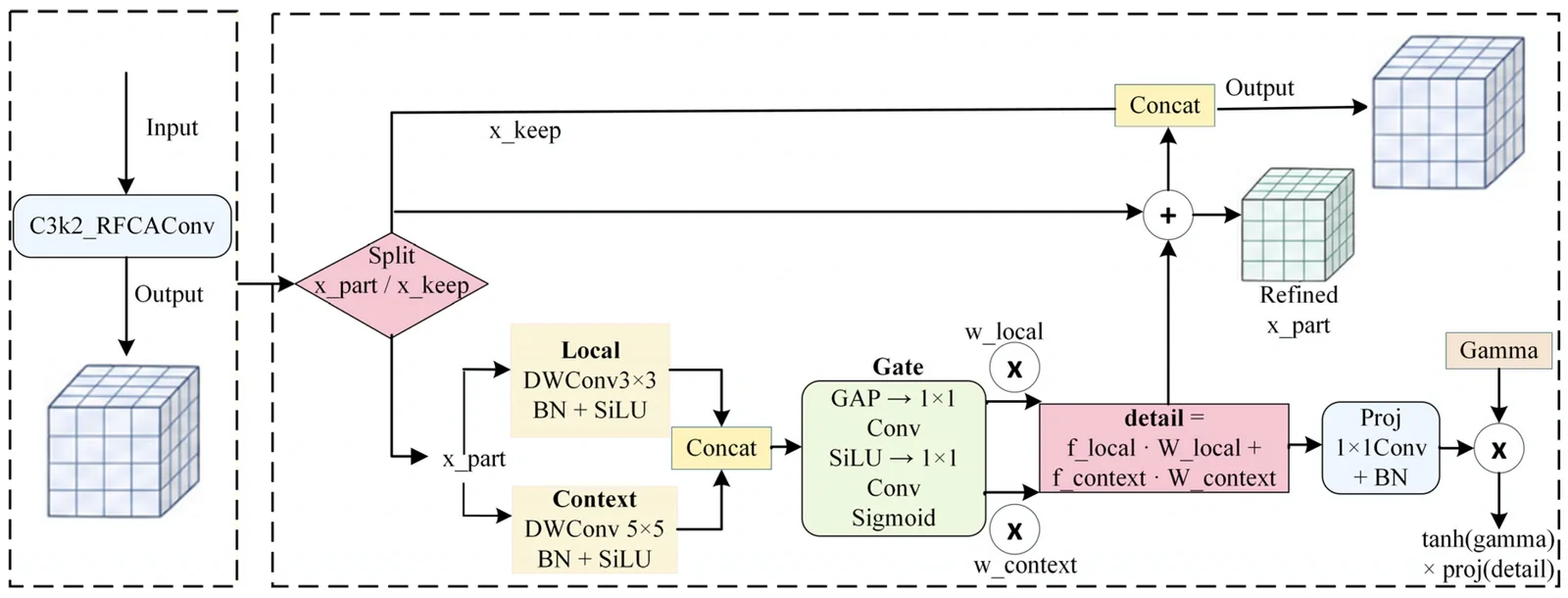
Structure of the LCA-C3k2 module.

To jointly model local edge details and neighborhood context information of maize lesions, this study designs the LCA-C3k2 local-context adaptive enhancement module. The structure of LCA-C3k2 is shown in Figure 10. The module uses C3k2_RFCAConv as the feature aggregation backbone and introduces a channel-selective local-context enhancement structure. Inspired by partial channel computation in Partial Convolution (PConv) [32], LCA-C3k2 reconstructs selected channels to reduce redundant feature processing and minimize disturbance to the original feature distribution. Unlike PConv, which mainly targets inference efficiency, LCA-C3k2 focuses on joint representation of edge details and neighborhood semantic information in multi-part maize disease scenarios.

Specifically, the input feature first passes through C3k2_RFCAConv for basic feature aggregation, after which selected channels enter the local-context enhancement mechanism. A 3 × 3 depthwise convolution captures lesion edges and fine-grained texture features, while a 5 × 5 depthwise convolution enlarges the local receptive field and models neighborhood context information. The two responses are dynamically fused using adaptive weights. The enhanced features are concatenated with the unenhanced channels, achieving collaborative enhancement of local details and contextual semantics while maintaining channel compatibility and original feature stability.

The process is expressed as follows:

(3) $$D=w_{l}\cdot F_{l}+w_{c}\cdot F_{c}$$

where D denotes the local-context fused feature, $F_{l}$ denotes the local detail feature obtained by 3 × 3 depthwise convolution, $F_{c}$ denotes the contextual feature obtained by 5 × 5 depthwise convolution, and $w_{l}$ and $w_{c}$ denote the adaptive selection weights of the local and contextual branches, respectively.

To avoid excessive disturbance of the original channel information by the enhanced features, LCA-C3k2 uses learnable parameters to regulate enhancement intensity and supplements the enhanced channels through a residual form. The final output process is expressed as:

(4) $$Y=C(X_{p}+tanh(\gamma )\cdot P(D),X_{k})$$

where Y denotes the output feature of LCA-C3k2, $X_{p}$ denotes the partial channel features involved in enhancement, $X_{k}$ denotes the directly retained channel features, P denotes the projection mapping operation, C denotes channel concatenation, γ denotes a learnable scaling parameter, and tanh controls the intensity of local-context enhanced feature injection.

Through partial channel enhancement and local-context adaptive fusion, LCA-C3k2 improves representation of maize disease fine-grained textures, edge structures, and local context information while maintaining channel-structure stability.

#### 2.5.3. GRN-BiAttn (Global-Response-Normalized Binary Attention)

**Figure 11:**
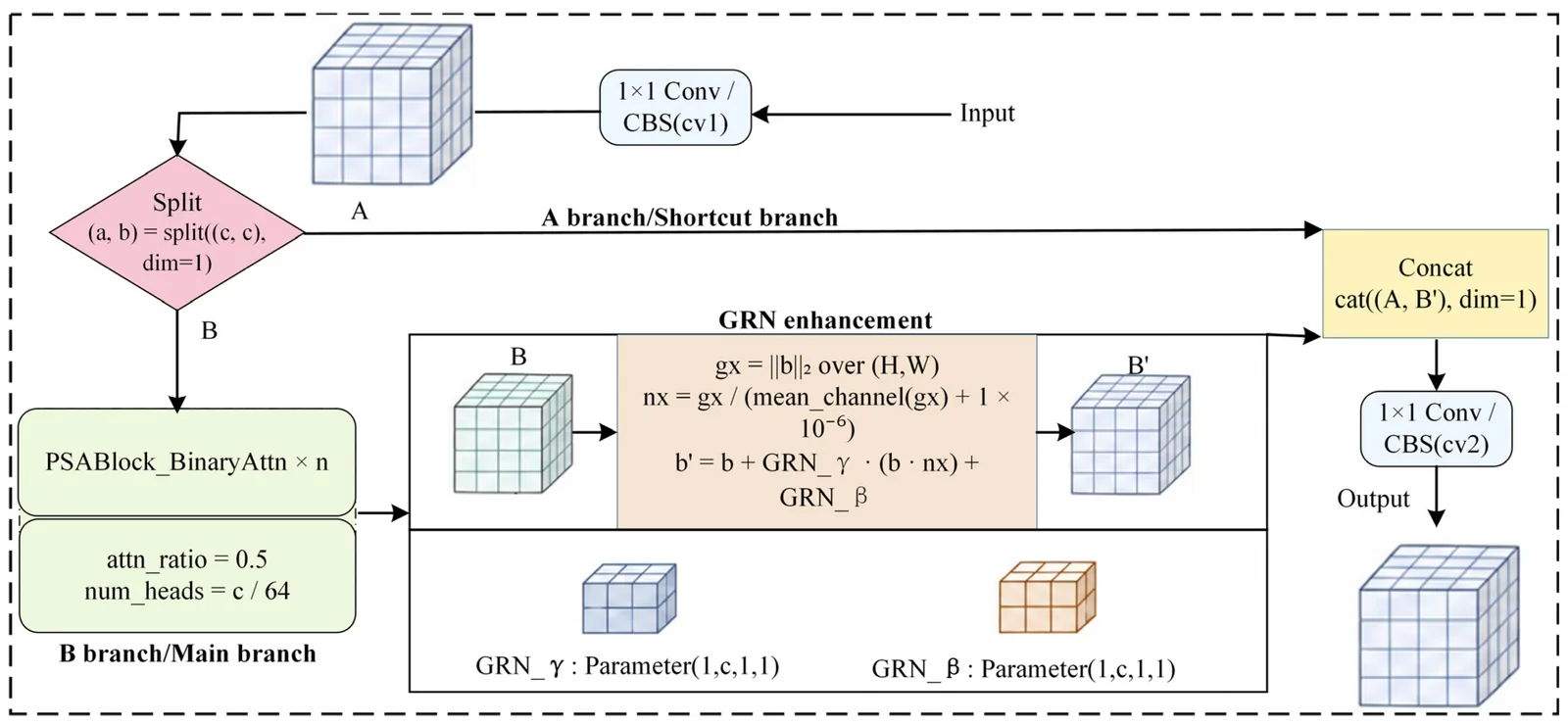
Structure of the GRN-BiAttn module.

To enhance representation of key semantic regions related to maize diseases, this study designs the GRN-BiAttn global-response-normalized binary attention module. The module reconstructs the attention modeling path and introduces a binary attention branch (PSABlock_BinaryAttn) to improve selective representation of disease-related semantic regions. Existing studies show that global response normalization can adaptively regulate response intensity across channels [33]. On this basis, a channel-response calibration unit is further designed to regulate binary-attention-enhanced semantic features through global response normalization and suppress redundant feature responses from complex backgrounds. Compared with a single attention enhancement strategy, GRN-BiAttn improves stability and discriminability of key disease regions in high-level semantic features through the joint effects of binary attention modeling and global response normalization. The structure of GRN-BiAttn is shown in Figure 11.

GRN-BiAttn first divides the input feature into a retained branch and an attention branch through input mapping convolution. The retained branch directly participates in subsequent feature fusion, while the attention branch is fed into PSABlock_BinaryAttn for semantic feature extraction. Let the output feature of the attention branch be B; the module computes its L2-norm response over the spatial dimensions and normalizes it using the channel mean.

The computation is defined as:

(5) $$G=||B|{|}_{2},\;\;\;N=\frac{G}{M(G)+\varepsilon }$$

where G denotes the global response of the attention-branch feature, N denotes the normalized channel response weight, M denotes the mean operation along the channel dimension, and ε is a constant that prevents the denominator from being zero.

The module then uses learnable parameters to regulate the attention feature through global response normalization, expressed as:

(6) $$B^{\prime }=B+\gamma \cdot (B\cdot N)+\beta$$

where $B^{\prime }$ denotes the feature after global response regulation, B denotes the attention-branch output feature, N denotes the normalized channel response weight, and γ and β denote learnable scaling and bias parameters, respectively.

Through binary attention modeling and global response normalization, GRN-BiAttn enhances channel responses in key semantic regions and suppresses redundant features in complex backgrounds, thereby improving the discriminative stability of high-level semantic features.

## 3. Results

### 3.1. Experimental Platform

The experiments were conducted on Ubuntu 22.04. The experimental platform was equipped with 10 vCPUs, 60 GB of memory, and an NVIDIA GeForce RTX 4090 GPU with 24 GB of video memory. Model training and testing were performed using the PyTorch deep learning framework, with PyTorch 25.03-Ubuntu22.04 as the development environment. This hardware platform met the computational requirements for training, validation, and testing of YOLOv11-MPD and provided a stable environment for subsequent comparative experiments on maize disease detection. All experiments used the parameter settings listed in Table 2.

### 3.2. Evaluation Metrics

For model performance evaluation, this study mainly used Precision (*P*), Recall (*R*), *mAP50*, *mAP50-95*, the number of parameters (Params, M), and model weight size (MB). Precision and Recall evaluate the accuracy and completeness of maize disease target detection. *mAP50* represents mean average precision at an intersection over union (IoU) threshold of 0.50, whereas *mAP50-95* represents average detection precision over IoU thresholds ranging from 0.50 to 0.95. The number of parameters and model size are used to evaluate lightweight performance.

(7) $$P=\frac{TP}{TP+FP}$$

(8) $$R=\frac{TP}{TP+FN}$$

(9) $$mAP_{50}=\frac{1}{C}\sum_{c=1}^{C}AP_{c,50}$$

(10) $$mAP_{50-95}=\frac{1}{10C}\sum_{i=1}^{10}\sum_{c=1}^{C}{AP}_{c,i}$$

where TP denotes the number of correctly detected targets, FP denotes the number of false detections, FN denotes the number of missed detections, C denotes the number of classes, $AP_{c,50}$ denotes the average precision of class c at an IoU threshold of 0.50, and ${AP}_{c,i}$ denotes the average precision of class c at an IoU threshold of i.

### 3.3. Comparison with Different Models

To verify the overall performance of YOLOv11-MPD in multi-part maize disease detection, this study compared it with representative object detection models, including YOLOv5, YOLOv6, YOLOv8, YOLOv10n, YOLOv11n, YOLOv12, YOLOv26 [34], Faster R-CNN, SSD, and DEIM. The results are shown in Table 3. All models used the same data split, input size, and training parameter settings. For the comparison reported in Table 3, each model was trained once under these fixed settings. To further assess the influence of random variation on the training results of the proposed model, YOLOv11-MPD was independently trained using random seeds of 0, 1, and 2, and the resulting variation in mAP50, together with the mean and standard deviation, is reported in Section 3.4.

As shown in Table 3, YOLOv11-MPD achieved Precision, Recall, mAP50, and mAP50-95 of 72.3%, 72.8%, 79.5%, and 50.2%, respectively. Compared with the YOLOv11n baseline, YOLOv11-MPD improved Precision, Recall, mAP50, and mAP50-95 by 2.4, 2.5, 2.9, and 2.4 percentage points, respectively, while reducing the number of parameters from 2.6 M to 2.4 M and maintaining a similar model weight size. These results indicate that the proposed improvements enhance detection accuracy while keeping the model compact. Compared with YOLOv8, YOLOv12, and YOLOv26, YOLOv11-MPD obtained the best Recall, mAP50, and mAP50-95 among YOLO-series models, showing stronger overall performance in reducing missed detections and improving localization accuracy. Across different detection frameworks, YOLOv11-MPD improved Precision by 25.3 percentage points and reduced parameters and model size by approximately 98.2% and 95.1%, respectively, compared with Faster R-CNN. Compared with SSD, YOLOv11-MPD improved Recall and mAP50 by 52.4 and 10.7 percentage points, respectively, while using substantially fewer parameters and a smaller model size. Compared with DEIM, YOLOv11-MPD improved Recall, mAP50, and mAP50-95 by 1.1, 0.3, and 2.1 percentage points, respectively, while reducing the parameter count and model weight size by approximately 35.5% and 63.4%, respectively.

Although some models achieved higher values for individual metrics, YOLOv11-MPD maintained high and balanced performance across Precision, Recall, mAP50, and mAP50-95 with only 2.4 M parameters. Its advantage was not achieved by sacrificing other metrics; instead, it preserved strong overall detection performance with a compact model.

### 3.4. Training Stability Under Different Random Seeds

To evaluate the training stability of YOLOv11-MPD under the current experimental setting, three independent training runs were conducted using random seeds of 0, 1, and 2. The dataset split and all other training settings were kept unchanged. As shown in Table 4, the mAP50 values obtained from the three runs were 79.5%, 79.3%, and 79.6%, respectively. The mean mAP50 was 79.47%, with a standard deviation of 0.15 percentage points. The small variation among the independent runs indicates that YOLOv11-MPD provides consistent mAP50 results under different random seeds within the current dataset and experimental setting.

### 3.5. Ablation Experiments

#### 3.5.1. Internal Ablation of the RFCAConv Family

To verify the detail compensation effect of the RFCAConv family, this study further introduced FG-RFCAConv on the basis of RFCAConv and conducted ablation comparisons. To better separate the individual contribution of FG-RFCAConv, an additional configuration using only FG-RFCAConv was added. The results are shown in Table 5.

As shown in Table 5, when only RFCAConv was introduced, the model achieved Precision, Recall, mAP50, and mAP50-95 of 73.3%, 71.4%, 77.7%, and 48.3%, respectively. When only FG-RFCAConv was used, the model obtained Precision, Recall, mAP50, and mAP50-95 of 72.5%, 67.9%, 76.6%, and 46.8%, respectively, with 2.6 M parameters and a model size of 5.2 MB. These results indicate that FG-RFCAConv alone does not provide a clear overall performance improvement, and its independent contribution is limited. After adding FG-RFCAConv to RFCAConv, Precision, mAP50, and mAP50-95 increased to 75.1%, 77.9%, and 48.9%, improving by 1.8, 0.2, and 0.6 percentage points over RFCAConv alone, while the number of parameters and model size remained 2.7 M and 5.4 MB. This result indicates a certain complementary effect between FG-RFCAConv and RFCAConv, with FG-RFCAConv mainly providing supplementary detail compensation during P3-to-P4 scale transfer. Therefore, FG-RFCAConv is better interpreted as a supplementary detail-compensation structure that works collaboratively with RFCAConv rather than as an independently effective enhancement module.

#### 3.5.2. Ablation of the C3k2 Family

To verify the influence of different structures in the C3k2 family on model performance, this study used C3k2_RFCAConv as the basis and introduced LCA-C3k2 and HGD-C3k2 for ablation comparisons. An additional configuration using HGD-C3k2 alone was included to evaluate its independent contribution. The results are shown in Table 6.

When only C3k2_RFCAConv was introduced, the model achieved Precision, Recall, mAP50, and mAP50-95 of 73.6%, 68.0%, 75.8%, and 47.0%, respectively. After adding LCA-C3k2, Precision, mAP50, and mAP50-95 increased to 76.7%, 76.3%, and 47.6%, respectively. When HGD-C3k2 was introduced alone, the four metrics reached 74.3%, 68.1%, 76.8%, and 47.3%, respectively, with mAP50 improving by 1.0 percentage point over C3k2_RFCAConv, indicating a certain independent contribution of HGD-C3k2.

When LCA-C3k2 and HGD-C3k2 were jointly introduced, mAP50 further increased to 77.8%, the highest among the four configurations, indicating a certain complementary effect between the two modules. Across all four experiments, the number of parameters and model size remained 2.6 M and 5.4 MB. Overall, LCA-C3k2 emphasizes local-context discrimination, whereas HGD-C3k2 mainly enhances small-scale details in the P3 branch.

#### 3.5.3. Overall Ablation

To verify the contribution of each improved structure to YOLOv11-MPD, YOLOv11n was used as the baseline. The RFCAConv family, C3k2 family, GRN-BiAttn, and Detect_LSDECD were introduced separately and in combination, and detection performance under different module combinations was evaluated. The results are shown in Table 7. All experiments used the same dataset split, input size, and training parameter settings to ensure comparability.

As shown in Table 7, different modules make distinct contributions to detection performance and model complexity. The RFCAConv family increased Precision from 69.9% to 75.1% and mAP50-95 from 47.8% to 48.9%, indicating that directional position awareness and detail compensation improve discrimination of diseased regions. The C3k2 family increased mAP50 to 77.8%, demonstrating enhanced feature aggregation and fine-grained texture representation. GRN-BiAttn increased Precision to 75.9%, reflecting its enhancement of key semantic regions. Detect_LSDECD increased mAP50-95 to 49.1% while reducing the number of parameters, indicating that the shared detail-enhanced detection head improves localization quality under high IoU thresholds. For module combinations, jointly using the RFCAConv and C3k2 families increased mAP50 to 79.8% and Recall to 72.6%, indicating complementarity in spatial position awareness and feature aggregation. After Detect_LSDECD was further added, Recall and mAP50-95 increased to 75.1% and 50.1%, respectively, although Precision decreased, suggesting that this detection head focuses more on improving target recall and localization quality. When all four types of improved structures were introduced, the final model achieved Precision, Recall, mAP50, and mAP50-95 of 72.3%, 72.8%, 79.5%, and 50.2%, respectively, with 2.4 M parameters. Overall, YOLOv11-MPD improves multi-scale disease detection performance while maintaining low model complexity, verifying the collaborative effectiveness of the modules in feature extraction, feature fusion, and detection prediction.

These results indicate that the improved modules are not simply stacked independently but provide complementary effects at different network stages. The RFCAConv family and C3k2 family jointly enhance spatial-position perception and fine-grained feature aggregation, leading to a clear improvement in mAP50. Detect_LSDECD improves Recall and mAP50-95, indicating better consistency of multi-scale prediction features. GRN-BiAttn suppresses redundant background responses and improves overall detection stability. From the perspective of the performance–complexity trade-off, the current ablation results show that different module combinations provide different balances between detection performance and model complexity. For example, the RFCAConv family + C3k2 family combination achieved a relatively high mAP50, while the addition of Detect_LSDECD further increased Recall and mAP50-95.

#### 3.5.4. Functional Positioning of the Improved Modules

Table 8 provides a comprehensive summary of the main improved modules in YOLOv11-MPD, including their purpose, relative computational cost, and contribution to the overall improvement. As shown in Table 7, each module has a clear design objective and a relatively independent functional role, addressing a specific problem at a different network stage rather than repeatedly optimizing the same function. Each module can independently perform its corresponding task, such as feature enhancement, information compensation, semantic regulation, or detection prediction optimization, while forming complementary relationships with the other modules. Therefore, the structural design of YOLOv11-MPD is not a simple stacking of multiple modules but a coordinated architecture designed for multi-part maize disease detection.

### 3.6. Visual Results

#### 3.6.1. Qualitative Visualization Comparison

**Figure 12:**
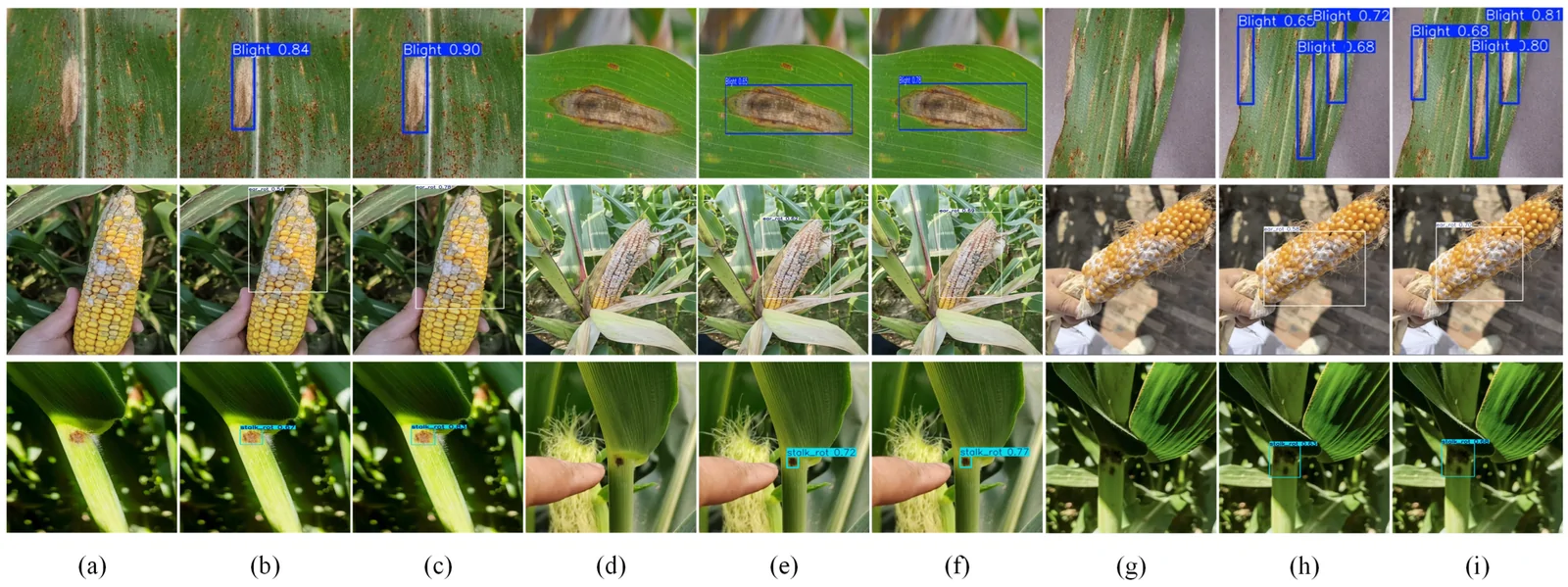
Comparison of detection results. (**a**,**d**,**g**) Original images; (**b**,**e**,**h**) detection results of YOLOv11n; (**c**,**f**,**i**) detection results of YOLOv11-MPD.

Figure 12 presents qualitative visualization examples of YOLOv11n and YOLOv11-MPD on maize leaf, ear, and stalk disease samples. The selected samples include different maize organs and field scenes with variations in lesion size, illumination, partial occlusion, and background complexity.

For the selected leaf disease samples, both models detected the main lesion regions, while YOLOv11-MPD produced more complete bounding-box coverage and higher confidence scores in several examples. For the ear disease samples, the irregular kernel structure and surrounding leaves increased the difficulty of lesion localization; compared with YOLOv11n, YOLOv11-MPD showed more consistent coverage of the diseased regions in the selected examples. For the stalk disease samples, where the diseased regions were relatively small and the background interference was more evident, YOLOv11-MPD provided more focused detection boxes for the lesion areas. These visualization results provide qualitative evidence based on representative samples, while the overall conclusions regarding localization performance and background-interference resistance are mainly supported by the quantitative results reported in the experimental tables.

#### 3.6.2. Training-Curve Visualization Comparison

**Figure 13:**
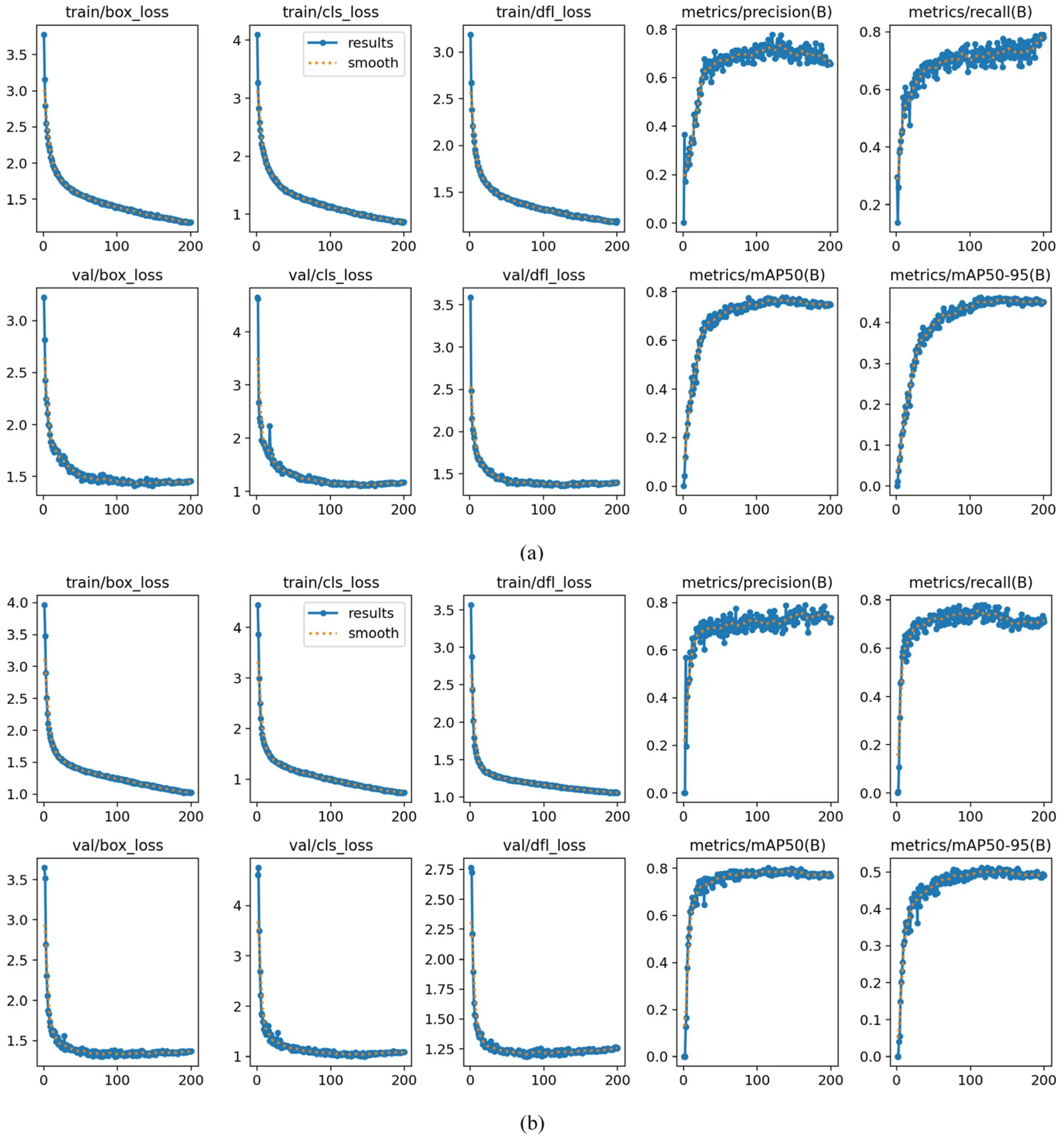
Comparison of training curves. (**a**) YOLOv11n; (**b**) YOLOv11-MPD. Each panel includes training box loss, training classification loss, training DFL loss, Precision, Recall, validation box loss, validation classification loss, validation DFL loss, mAP50, and mAP50-95 curves.

To analyze training stability of YOLOv11-MPD in multi-part maize disease detection, this study compared its training losses and evaluation metric curves with those of the baseline YOLOv11n. As shown in Figure 13, the losses of both models decreased rapidly at the initial training stage and then gradually stabilized. At the same time, Precision, Recall, mAP50, and mAP50-95 generally increased and remained relatively stable in the later stage, indicating that the models progressively learned effective maize disease target features.

The training and validation losses of YOLOv11-MPD were smoother, without obvious oscillation or divergence, and its mAP50 and mAP50-95 curves remained at higher levels in the later training stage compared with YOLOv11n. These results indicate that the improved model has good training stability and detection performance for maize diseases from different plant parts. They are consistent with the quantitative results and further verify the effectiveness of YOLOv11-MPD for multi-part maize disease detection on this dataset.

#### 3.6.3. Error Analysis

**Figure 14:**
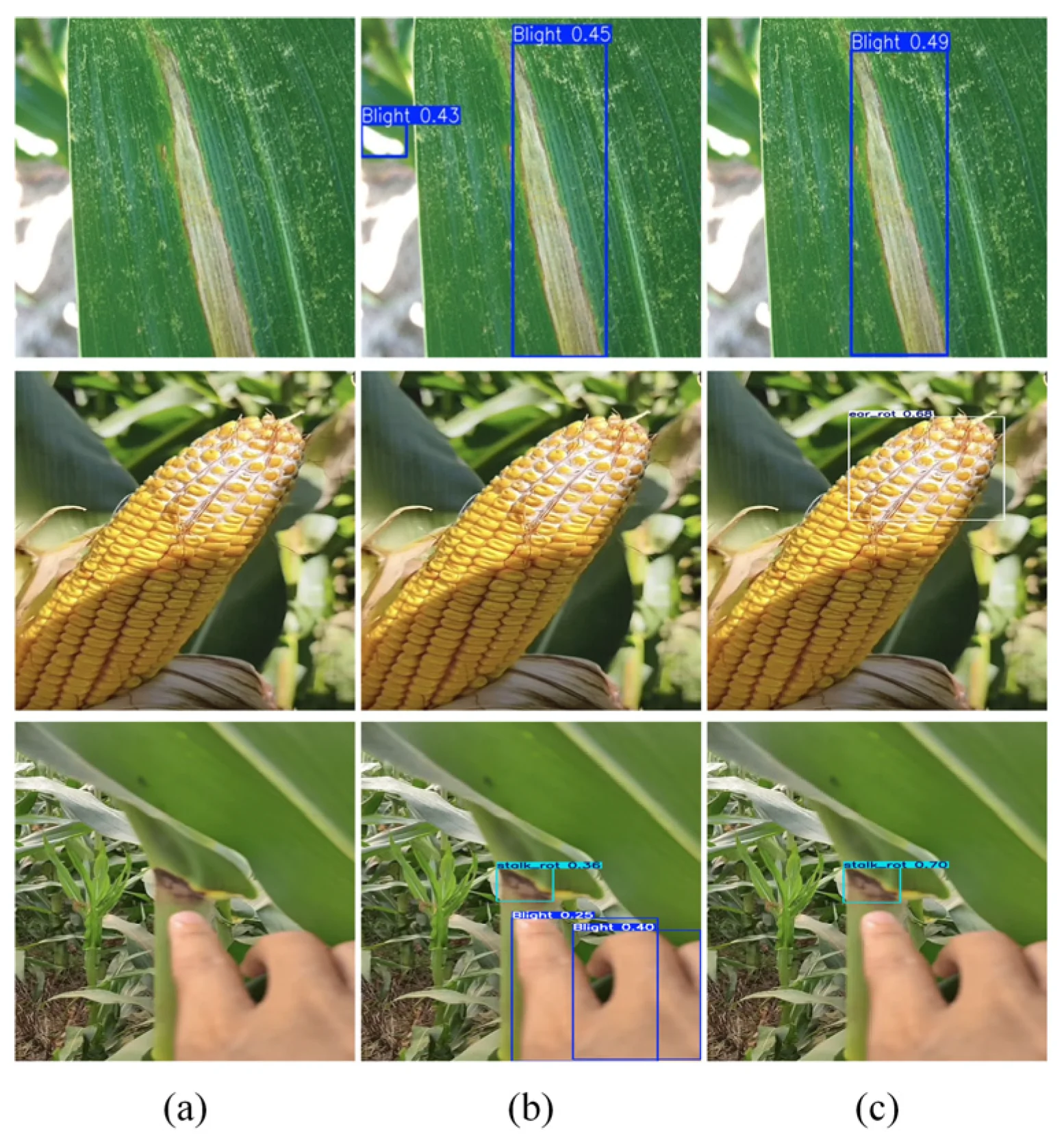
Typical challenging cases and error analysis of maize disease detection. (**a**) Original images; (**b**) detection results of YOLOv11n; (**c**) detection results of YOLOv11-MPD.

To analyze the error sources of the model, typical challenging samples from the test set were selected for visual analysis, as shown in Figure 14. False positives mainly occurred in regions where background textures, highlighted leaf edges, or plant tissue patterns were similar to lesion features, while false negatives mainly appeared in samples with small lesions, blurred boundaries, or weak textures. In addition, imaging conditions also affected detection performance. Drastic illumination changes could reduce the contrast between diseased and healthy tissues, long shooting distances made lesions smaller, and leaf or organ overlap could lead to incomplete lesion boundaries, thereby affecting detection stability. Compared with YOLOv11n, YOLOv11-MPD showed better detection performance under strong background interference, small lesions, weak-texture boundaries, and occlusion conditions, reducing some false positives and false negatives and improving the localization stability of diseased regions.

### 3.7. Edge Deployment on Raspberry Pi

**Figure 15:**
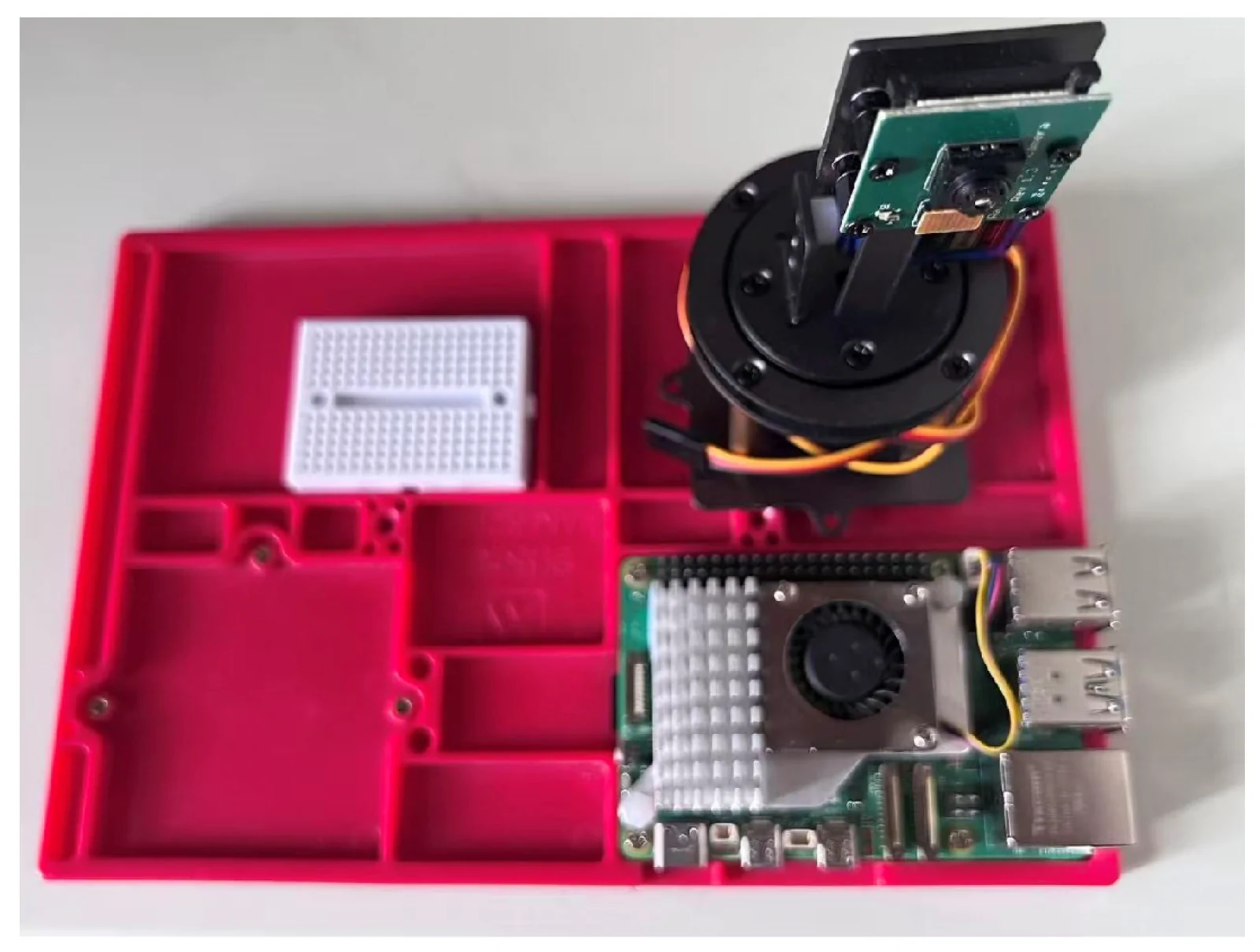
Raspberry Pi edge deployment platform.

**Figure 16:**
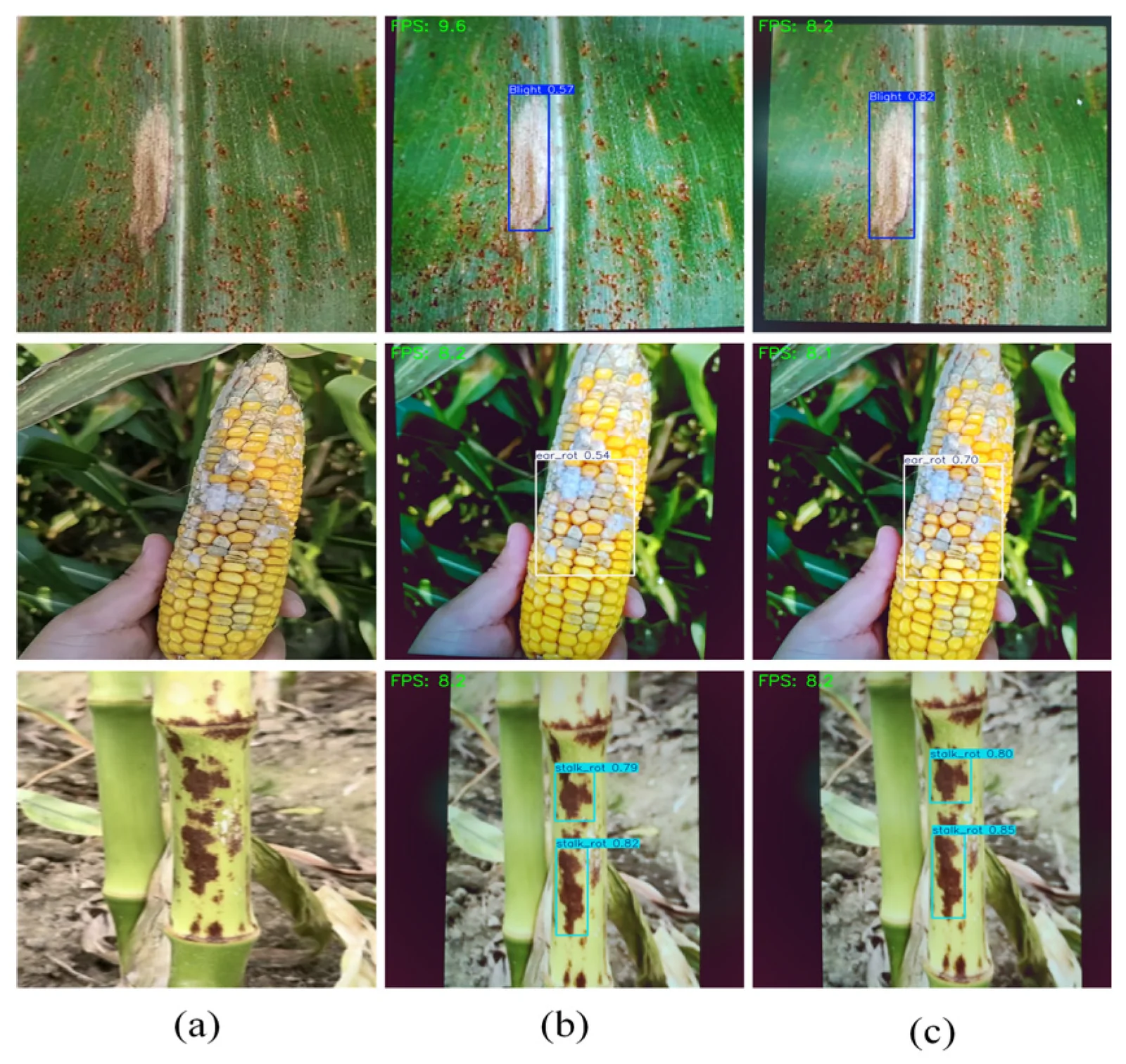
Detection examples on the Raspberry Pi platform. (**a**) Original images; (**b**) detection results of YOLOv11n; (**c**) detection results of YOLOv11-MPD.

To examine whether YOLOv11-MPD can execute single-frame image inference on a resource-limited edge platform under the current experimental setting, the trained model was deployed on a Raspberry Pi 5. The edge device was equipped with 16 GB of memory. To maintain consistency with the offline testing setting, the input image size was fixed at 640 × 640, and the trained model was converted into ONNX format for inference. YOLOv11n and YOLOv11-MPD were selected as the compared models and tested under the same hardware platform, input size, model format, and inference precision. The physical Raspberry Pi deployment device is shown in Figure 15, and representative detection examples are shown in Figure 16.

The deployment results show that YOLOv11-MPD can perform multi-part maize disease detection on the Raspberry Pi 5 platform. Compared with YOLOv11n, YOLOv11-MPD maintains a compact model size and produces higher prediction confidence for diseased regions, indicating that the improved model can still preserve stronger disease feature representation under edge-side inference conditions. Although the edge-side inference speed does not show a clear advantage, YOLOv11-MPD can still complete single-frame inference on the Raspberry Pi 5 under the current deployment configuration. This experiment demonstrates technical executability on the tested device rather than general field-deployment capability. Future work will further consider model pruning, quantization compression, and knowledge distillation to improve inference efficiency and real-time detection performance on resource-limited devices.

## 4. Discussion

The experimental results demonstrate that YOLOv11-MPD achieves balanced overall performance in multi-part maize disease detection. Compared with YOLOv11n, YOLOv11-MPD improves Precision, Recall, mAP50, and mAP50-95 while reducing the number of parameters from 2.6 M to 2.4 M, indicating that the proposed structural modifications improve disease feature representation without increasing model scale. Maize leaf, stalk, and ear diseases differ substantially in lesion scale, morphology, and texture distribution, while illumination variation, shooting viewpoint, and natural background interference further increase detection difficulty. Therefore, collaborative enhancement at multiple feature levels is important for maintaining stable detection performance across different maize organs.

The ablation experiments indicate that the improved structures provide complementary effects at different network stages. Multi-part maize diseases exhibit substantial scale variation, while some small and low-contrast lesions have weak boundaries and indistinct textures, making their responses susceptible to attenuation during downsampling and cross-scale feature transfer. Meanwhile, leaf veins, stalk textures, soil, and surrounding vegetation may produce local responses similar to lesion features, increasing the risk of false detections. To address these characteristics, HGD-C3k2 and FG-RFCAConv enhance high-resolution details in P3 and compensate for information loss during P3-to-P4 feature transfer, respectively; LCA-C3k2 integrates local details with neighborhood context; GRN-BiAttn regulates feature responses under complex backgrounds; and Detect_LSDECD improves the consistency of multi-scale detection features. Therefore, this structural complementarity, ranging from detail preservation and cross-scale compensation to context modeling and multi-scale prediction, provides a plausible explanation for the improved detection performance of YOLOv11-MPD for small and low-contrast disease symptoms on the current dataset.

Relative to the representative models evaluated in Table 3, the main distinction of YOLOv11-MPD is not an increase in model capacity but a task-driven collaborative design for multi-part maize diseases. Compared with Faster R-CNN, YOLOv11-MPD maintains a substantially smaller parameter count and model weight size while providing more balanced overall detection performance. Compared with other lightweight YOLO models, YOLOv11-MPD introduces targeted enhancement for high-resolution details, cross-scale information transfer, local-context relationships, background-response calibration, and multi-scale prediction. Therefore, its performance improvement is not obtained by optimizing a single metric or sacrificing other evaluation indicators but by improving complementary feature representations across different stages of the detection network.

This study still has some limitations. On the one hand, the high-frequency detail enhancement structures may activate plant or soil textures while strengthening small-lesion responses, which can produce false-positive responses and fluctuations in individual metrics. On the other hand, the current evaluation was conducted only on the constructed multi-part maize disease dataset, without independent external validation covering all three target categories. Most currently available public maize disease datasets mainly contain leaf images, whereas object-detection datasets for maize ear and stalk diseases remain limited. Therefore, the conclusions should be interpreted within the scope of the dataset used in this study and do not fully demonstrate broader generalization across regions, growth stages, climatic conditions, disease types, or imaging devices. A potential reason for performance degradation when transferring the model to an independent dataset is the distribution shift between the training and target domains. Differences in regions and weather conditions may alter illumination, leaf-surface reflectance, background color, and lesion visibility, while variations in camera sensors, resolution, color response, and image quality can further change image appearance. Moreover, the current training samples mainly consist of typical late-stage disease images collected under specific acquisition conditions, which may cause the model to learn texture, color, and background characteristics associated with the current data distribution. When an independent dataset differs substantially in disease severity, imaging conditions, or background composition, these domain shifts may alter feature responses and consequently reduce detection performance. Another unresolved issue is scalability, the current study considers only three maize disease categories, and it remains unclear whether YOLOv11-MPD can maintain its current performance advantage when the number of disease classes or the dataset scale increases substantially. A larger number of categories may increase inter-class similarity and feature confusion, while larger and more diverse datasets may alter the training and feature-learning characteristics of the model. Therefore, the scalability of the proposed architecture to more complex class settings and substantially larger datasets requires further validation. From the perspective of practical crop monitoring, YOLOv11-MPD could serve as a detection component within a field image acquisition and disease recognition pipeline. However, its practical performance may still be affected by changes in agronomic conditions, maize varieties, plant developmental stages, and planting patterns. These factors can alter plant morphology, background structure, lesion appearance, and target scale, resulting in distribution differences from the current training data. Therefore, further validation across more varieties, growth stages, planting densities, and field management conditions is required before integrating the model into practical crop monitoring systems. Regarding edge deployment, the Raspberry Pi 5 experiment provides only a preliminary evaluation based on single-frame image inference and does not assess long-term operational stability, power consumption, continuous video-stream processing performance, or the influence of different edge hardware platforms. Therefore, the current edge deployment results should not be regarded as a substitute for a comprehensive evaluation of the model’s operational characteristics. Future work will further investigate cross-domain data expansion, external validation, video-stream testing, power consumption and long-term stability, multi-platform deployment, model compression, and inference acceleration to further evaluate and improve the generalization and practical applicability of YOLOv11-MPD.

## 5. Conclusions

This study addressed multi-part maize disease detection on leaves, stalks, and ears by constructing a multi-source maize disease image dataset and proposing the YOLOv11-MPD detection model. To address large target-scale variation, weak fine-grained texture, and strong complex-field background interference, the model introduces spatial position awareness, shallow detail enhancement, local-context modeling, semantic response regulation, and lightweight detection-head design, thereby improving representation of lesion edges, small targets, and key semantic regions. The experimental results show that on the dataset constructed in this study, YOLOv11-MPD, achieves Precision, Recall, mAP50, and mAP50-95 of 72.3%, 72.8%, 79.5%, and 50.2%, respectively, improving YOLOv11n by 2.4, 2.5, 2.9, and 2.4 percentage points while reducing parameters from 2.6 M to 2.4 M. Comparative experiments, ablation experiments, and visualization results demonstrate improved detection performance of YOLOv11-MPD within the dataset and experimental settings used in this study. The Raspberry Pi 5 experiment further shows that the model can perform single-frame edge-side inference under the tested configuration. These results mainly reflect the model performance under the current experimental conditions, while its applicability to continuous operation and industrial scenarios still requires further validation across more diverse datasets, field environments, and deployment platforms.

## Figures and Tables

**Table 1:** Statistical information of the maize disease dataset after data splitting and training-set augmentation.

Disease Category	Original Images	Training Images After Augmentation	Validation Images	Test Images	Bounding-Box Instances	Average Objects per Image
Blight	658	700	67	65	3430	4.12
Ear_rot	706	700	67	66	4396	5.28
Stalk_rot	669	700	71	71	4800	5.70
Total	2033	2100	205	202	12,626	5.04

**Table 2:** Training parameter settings.

Parameter	Setting
Epoch	200
Batch size	32
Image size	640 × 640
Momentum	0.937
Initial learning rate	0.01
Workers	8
Optimizer	SGD
Weight decay	0.0005
Learning rate scheduler	Linear decay

Note: The training epochs, initial learning rate, and optimizer listed in Table 2 were adopted from the default training configuration of the YOLO framework. To ensure a fair comparison, the same training settings were used for all compared and ablation models. The validation set was used only to monitor training and select the best-performing weights, rather than for hyperparameter optimization.

**Table 3:** Comparative experimental results for different detection models.

Model	Precision (%)	Recall (%)	mAP50 (%)	mAP50-95 (%)	Params (M)	Weight Size (MB)
YOLOv5	64.2	68.1	72.5	42.5	2.5	5.0
YOLOv6	67.9	66.6	74.7	46.4	4.2	8.3
YOLOv8	73.9	63.5	74.5	46.2	3.0	6.0
YOLOv10n	68.1	64.0	70.7	42.4	2.3	5.5
YOLOv11n	69.9	70.3	76.6	47.8	2.6	5.2
YOLOv12	74.2	64.8	76.3	47.0	2.6	5.3
YOLOv26	71.2	63.2	74.1	46.8	2.4	5.1
Faster R-CNN	47.0	94.1	85.1	-	137.1	108.0
SSD	79.5	20.4	68.8	-	26.3	100.3
DEIM	72.3	71.7	79.2	48.1	3.72	14.5
YOLOv11-MPD	72.3	72.8	79.5	50.2	2.4	5.3

Note: mAP50-95 refers to the COCO-standard mean average precision over multiple IoU thresholds. Faster R-CNN and SSD are natively evaluated under the PASCAL VOC protocol, and their default frameworks output only mAP at IoU = 0.5; the dash indicates that mAP50-95 was not additionally calculated for these models in this study.

**Table 4:** Training stability of YOLOv11-MPD under different random seeds.

Model	Random Seed	mAP50 (%)
YOLOv11-MPD	0	79.5
YOLOv11-MPD	1	79.3
YOLOv11-MPD	2	79.6
Mean ± SD	—	79.47 ± 0.15

Note: The results are reported as the mean ± standard deviation of mAP50 over three independent training runs. The dataset split and all other training settings were kept unchanged, with only the random seed varied.

**Table 5:** Ablation experiment for the RFCAConv family.

RFCAConv	FG-RFCAConv	Precision (%)	Recall (%)	mAP50 (%)	mAP50-95 (%)	Params (M)	Weight Size (MB)
√	×	73.3	71.4	77.7	48.3	2.7	5.4
×	√	72.5	67.9	76.6	46.8	2.6	5.2
√	√	75.1	70.4	77.9	48.9	2.7	5.4

Note: “√” indicates that the corresponding module is included in the model, whereas “×” indicates that the corresponding module is not included.

**Table 6:** Ablation experiment for the C3k2 family.

C3k2_RFCAConv	LCA-C3k2	HGD-C3k2	Precision (%)	Recall (%)	mAP50 (%)	mAP50-95 (%)	Params (M)	Weight Size (MB)
√	×	×	73.6	68.0	75.8	47.0	2.6	5.4
√	√	×	76.7	67.4	76.3	47.6	2.6	5.4
√	×	√	74.3	68.1	76.8	47.3	2.6	5.4
√	√	√	74.1	68.6	77.8	47.5	2.6	5.4

**Table 7:** Overall ablation experiment.

RFCAConv Family	C3k2 Family	GRN-BiAttn	Detect_LSDECD	Precision (%)	Recall (%)	mAP50 (%)	mAP50-95 (%)	Params (M)	Weight Size (MB)
×	×	×	×	69.9	70.3	76.6	47.8	2.6	5.2
√	×	×	×	75.1	70.4	77.9	48.9	2.7	5.4
×	√	×	×	74.1	68.6	77.8	47.5	2.6	5.4
×	×	√	×	75.9	68.2	78.4	48.2	2.6	5.2
×	×	×	√	75.8	68.7	78.1	49.1	2.3	4.9
√	√	×	×	74.8	72.6	79.8	48.8	2.7	5.6
√	√	×	√	69.1	75.1	78.3	50.1	2.4	5.3
√	√	√	√	72.3	72.8	79.5	50.2	2.4	5.3

**Table 8:** Summary comparison of the improved modules in YOLOv11-MPD.

Module	Main Purpose	Relative Computational Cost	Contribution to Overall Improvement
RFCAConv	Enhances directional texture and spatial-position awareness	Moderate	Improves the basic representation of disease-related spatial and texture features
FG-RFCAConv	Compensates for detail loss during P3-to-P4 scale transfer	Low	Reduces information loss during downsampling and improves feature continuity across scales
HGD-C3k2	Enhances high-frequency details in the P3 high-resolution branch	Low	Strengthens the response to small lesions and weak-texture disease regions
LCA-C3k2	Performs local-context adaptive feature aggregation	Moderate	Improves the modeling of local edge information and neighboring contextual cues
GRN-BiAttn	Regulates semantic responses and suppresses redundant background interference	Low	Enhances key disease-related regions and improves response stability under complex backgrounds
Detect_LSDECD	Constructs a shared lightweight multi-scale detection head	Low/parameter-reducing	Improves classification and localization consistency while helping control the model scale

## Data Availability

Additional data generated during the current study are available from the corresponding author upon reasonable request.
